# *In* *silico* discovery of non-psychoactive scaffolds in Cannabis halting SARS-CoV-2 host entry and replication machinery

**DOI:** 10.2217/fvl-2021-0309

**Published:** 2022-04-04

**Authors:** Amira R Khattab, Mohamed Teleb

**Affiliations:** ^1^Pharmacognosy Department, College of Pharmacy, Arab Academy for Science, Technology & Maritime Transport, Alexandria, 1029, Egypt; ^2^Department of Pharmaceutical Chemistry, Faculty of Pharmacy, Alexandria University, Alexandria, 21521, Egypt

**Keywords:** ACE2, *Cannabis*, coronavirus, *in silico* modeling, M^pro^, SARS-CoV-2, spike protein

## Abstract

**Aim:** Coronavirus disease still poses a global health threat which advocates continuous research efforts to develop effective therapeutics. **Materials & methods:** We screened out an array of 29 cannabis phytoligands for their viral spike-ACE2 complex and main protease (M^pro^) inhibitory actions by *in silico* modeling to explore their possible dual viral entry and replication machinery inhibition. Physicochemical and pharmacokinetic parameters (ADMET) formulating drug-likeness were computed. **Results:** Among the studied phytoligands, cannabigerolic acid **(2)**, cannabigerol **(8)**, and its acid methyl ether **(3)** possessed the highest binding affinities to SARS-CoV-hACE2 complex essential for viral entry. Canniprene **(24)**, cannabigerolic methyl ether **(3)** and cannabichromene **(9)** were the most promising M^pro^ inhibitors. **Conclusion:** These non-psychoactive cannabinoids could represent plausible therapeutics with added-prophylactic value as they halt both viral entry and replication machinery.

## Graphical abstract

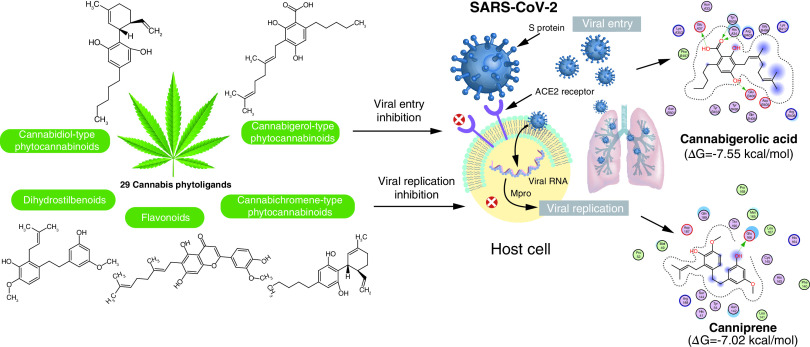

SARS-CoV-2 is the causative agent of raging pandemic of COVID-19 that is manifested by mild-to-severe respiratory tract inflammation leading to pneumonia, lung fibrosis and causing even death. Up till now there is no approved remedy for COVID-19, hence, many research endeavors have been directed toward finding therapeutic drug leads to combat the infection propagation. SARS-CoV-2 is a sense RNA virus bearing projections on its surface, i.e., envelope (E) and spike (S) glycoproteins with the latter initiating the infection via its attachment with host angiotensin-converting enzyme 2 (ACE2). ACE2 is an enzyme expressed in nasal and oral mucosa as well as lung and GI tract.

Nature provides a huge reservoir of anti-infectious phytochemicals with anti-inflammatory potential, from which many innovative phytotherapeutics were developed. Natural products and their metabolites in various plants were recently reported to possess striking inhibitory action against different SARS-CoV-2 proteins which could serve as valuable source of drug leads competent in combating COVID-19 pandemic [1]. Among which, polyphenols and flavonoids were reported to stop SARS-CoV-2 replication and thus, mitigating the infection propagation [2]. Natural therapeutics capable of blocking vital entry into host cells can represent a plausible therapeutic avenue with an added prophylactic action [3]. Emodin, tetra-*O*-galloyl-β-d-glucose and hesperidin are the only few S-protein-ACE2 binding inhibitors reported till now [4]. The virus releases its RNA into the host cells after its entry, then the viral genome codes for nonstructural proteins *viz.* main protease (M^pro^), RNA-dependent RNA polymerase, the structural glycoprotein and its helicase, and papain-like protease [4]. Inhibition of the M^pro^ activity is regarded as a second most important class of antiviral therapeutics as being able to inhibit viral replication, hence halting the infection spread [5]. Viral replication in host cells is well evidenced to be associated with hyper-induction of proinflammatory cytokines, known as ‘cytokine storm’ and immune activation which are the main pathogenic features of COVID-19 [6]. Oolonghomobisflavan-A, theasinensin-d, and theaflavin-3-*O*-gallate from tea were reported as M^pro^ inhibitors [7].

*Cannabis sativa* L. biosynthesizes an arsenal of resorcinyl core decorated scaffolds with para-oriented terpenyl, pentyl and isoprenyl groups collectively known as phytocannabinoids that possess a wide therapeutic spectrum *viz.* anti-cancer, anti-epileptic and analgesic actions [8].

Cannabis plants are divided into three different phenotypes according to the cannabinoid content: drug-type (phenotype I; *C. indica* Lam.) containing a higher proportion of THC, intermediate-type (phenotype II; *C. ruderalis* Janisch.) and fiber-type or hemp (phenotype III; *C. sativa* L.) with cannabidiol (CBD) as the main phytocannabinoid. The fiber-type *Cannabis* contains a very low Δ9-THC level, however it is rich in CBD and its related nonpsychoactive phytochemicals. This type has been used for textile manufacturing and food purposes but with no therapeutic actions. Various non-psychoactive phytocannabinoids are present in fiber-type cannabis such as cannabinoic acids *viz.* cannabigerolic acid (CBGA) and cannabidiolic acid (CBDA), and their decarboxylated forms, i.e., cannabigerol (CBG) and cannabidiol (CBD), in addition to other minor cannabinoids *viz.* cannabichromene (CBC), cannabinol (CBN), cannabichromenic acid (CBCA), cannabinolic acid (CBNA) and several phenolic compounds such as cannflavins A and B, and canniprene [9]. The phytocannabinoidal content decreases from the upper to the lower part of the plants, i.e., hemp flowers contain the highest level, followed by the upper leaves. The peak concentration of cannabinoids is attained during the growing periods of the plant (10–11 weeks after cultivation) [10].

In continuation to previous research work [4,11,12], we aimed in the current study to explore the relative dual prophylactic/therapeutic potential of 29 cannabis phytochemicals by *in silico* exploration of their inhibitory action on viral spike-ACE2 complex, i.e., essential for the viral entry, as well as the viral replication machinery via the M^pro^. Further, physicochemical and pharmacokinetic parameters (ADMET) formulating drug-likeness were computed.

## Materials & methods

### Phytoligands under study

An array of 29 phytoligands from *C. sativa* belonging to different classes *viz.* phytocannabinoids, stilbenoids and flavonoids were included in the study, i.e., cannabidiolic acid (CBDA) **(1)**, cannabigerolic acid (CBGA) **(2)**, cannabigerolic acid monomethyl ether **(3)**, cannabigerovarinic acid **(4)**, cannabigerovarin **(5)**, cannabidiol (CBD) **(6)**, cannabidivarinic acid **(7)**, cannabigerol (CBG) **(8)**, cannanbichromene (CBC) **(9)**, cannabivarin **(10)**, Δ9-tetrahydrocannabivarin (Δ^9^-THCV) **(11)**, cannabidivarin (CBDV) **(12)**, (+)-cannabichromenic acid **(13)**, cannabinolic acid **(14)**, cannabidinodiol (CBND) **(15)**, cannabinol methyl ether **(16)**, cannabinol **(17)**, cannabicyclolic acid **(18)**, cannabicyclol **(19)**, cannabielsoin A **(20)**, cannabitriol **(21)**, cannflavin A **(22)**, cannflavin B **(23)**, canniprene **(24)**, cannabifuran **(25)**, dehydrocannabifuran **(26)**, cannabicitran **(27)**, cannabiripsol **(28)** and cannabimovone **(29)**. 2D structures of these different phytocannabinoids were downloaded as structure-data file (SDF) from PubChem (https://pubchem.ncbi.nlm.nih.gov).

### Molecular docking of the phytoligands

Molecular Operating Environment (MOE) software package version 2016.10 (Chemical Computing Group, Montreal, Canada) was used for molecular docking of the two viral proteins under study *viz.* SARS-CoV-2 S protein C-terminal domain (SARS-CoV-2-CTD) and M^pro^. SARS-CoV-2-CTD crystal structure complexed with human ACE2 (hACE2) and the coordinates of M^pro^ in complex with its inhibitor 2-cyclohexyl-*N*-(3-pyridyl)acetamide were retrieved from the protein data bank (PDB ID 6LZG and 5R84, respectively) [11,13–15]. The proteins were prepared employing the default “structure preparation” module settings, where hydrogen atoms were added, hydrogen bonds were optimized, atomic clashes were removed, and the crystal structures were refined. The docking protocol was adopted as previously reported in [16]. Docking results were presented as a list based on the S-scores with RMSD values below 2 Å and graphically showing the phytoligands interactions.

### *In silico* analysis of physicochemical properties & ADME profiling of the phytoligands

The physicochemical properties and ADME profiles of the studied phytoligands in human body were predicted using SwissADME calculation toolkit [17] and the online-server PreADMET (https://preadmet.bmdrc.kr/) [18].

## Results

### Molecular docking analysis of cannabis phytoligands within SARS-CoV-2 spike (S) protein–ACE2 complex

SARS-CoV-2 entry into the host cell is initiated by the spike (S) glycoprotein attachment [19] to hACE2 which proceeds by the cleavage of S protein by host cell proteases into S1 and S2 subunits for receptor recognition and cell membrane fusion, respectively. S1 is further subdivided into N-terminal domain (NTD) and C-terminal domain (CTD), which both function as a receptor-binding entity [20]. The S1 CTD of SARS-CoV-2 functions as the key region interacting with the hACE2 receptor. The crystal structure of SARS-CoV-2-CTD in complex with a single hACE2 molecule in asymmetric unit (PDB ID: 6LZG) was used in our study [13].

Docking simulations were used to explore the ability of the studied phytoligands in destabilizing the virus-enzyme complex or preventing its formation. We proposed that possible accommodation/fitting of such phytochemicals at the SARS-CoV-2-CTD-h2ACE interface and their possible interactions with the key amino acids of the complex may provide promising insights for the possible prophylactic action of the studied phytoligands.

The most suitable site for docking the phytoligands into the SARS-CoV-2-CTD-2hACE binding interface was localized using ‘site finder’ feature of MOE 2016.10. in the absence of a co-crystallized inhibitor at the interface of the studied complex by employing flexible docking mode using reference phytochemical viral entry inhibitor, i.e., hesperidin [21–23]. Most of the studied phytoligands were found to display moderate to promising binding affinities compared with hesperidin **(30)** (Table 1). Cannabigerolic acid (CBGA) **(2)** recorded the best binding affinity (-7.55 kcal/mol), followed by cannabigerol (CBG) **(8)**, cannabigerolic acid monomethyl ether **(3)**, cannabigerovarinic acid **(4)**, cannabichromene (CBC) **(9)** and cannabifuran **(25)** showing slightly less binding affinities ranging from -7.21 to -7.01 kcal/mol. The remaining phytoligands showed a moderate fitting (-6.93 to -5.99 kcal/mol).

**Table 1.** Results of docking simulations of the studied cannabis phytoligands within SARS-CoV-2 spike (S) protein–ACE2 complex.

No.	Name of phytoligands	ΔG^†^ (kcal/mol)	Interactions at the binding interface	
			hACE2 residues	SARSCoV-2-CTD residues
1	Cannabidiolic acid (CBDA)	-6.53	Glu37	No interaction
2	Cannabigerolic acid (CBGA)	-7.55	Glu37	Arg403, Glu406, Tyr505
3	Cannabigerolic acid monomethyl ether	-7.21	**His34**	No interaction
4	Cannabigerovarinic acid	-7.21	Glu37	Glu406
5	Cannabigerovarin	-6.45	No interaction	Glu406
6	Cannabidiol (CBD)	-6.19	**His34**	No interaction
7	Cannabidivarinic acid	-6.42	No interaction	No interaction
8	Cannabigerol (CBG)	-6.66	No interaction	No interaction
9	Cannabichromene (CBC)	-7.11	No interaction	No interaction
10	Cannabivarin	-6.29	**His34**	No interaction
11	Δ9-Tetrahydrocannabivarin (Δ^9^-THCV)	-5.99	**His34**	No interaction
12	Cannabidivarin (CBDV)	-5.99	No interaction	No interaction
13	(+)-Cannabichromenic acid	-6.33	No interaction	Gln409**, Lys417**
14	Cannabinolic acid	-6.64	**His34**	Arg403
15	Cannabidinodiol (CBND)	-6.49	Glu37	No interaction
16	Cannabinol methyl ether	-6.65	No interaction	No interaction
17	Cannabinol	-6.53	Glu37	No interaction
18	Cannabicyclolic acid	-6.73	No interaction	No interaction
19	Cannabicyclol	-6.75	**His34**	No interaction
20	Cannabielsoin A	-6.81	No interaction	No interaction
21	Cannabitriol	-6.93	No interaction	Glu406
22	Cannflavin A	-6.55	**His34**	No interaction
23	Cannflavin B	-6.62	**His34**	No interaction
24	Canniprene	-6.67	No interaction	No interaction
25	Cannabifuran	-7.01	**His34**	No interaction
26	Dehydrocannabifuran	-6.83	**His34**	Arg403
27	Cannabicitran	-6.50	No interaction	No interaction
28	Cannabiripsol	-6.80	**His34**	No interaction
29	Cannabimovone	-6.93	No interaction	No interaction
30	Hesperidin	-7.51	**His34**	**Lys417**

The key residues involved in the SARS-CoV-2-CTD-2hACE complex formation are listed in bold.

† The ligand–receptor complex binding free energy at RMSD ≤2 Å.

### Molecular docking analysis of cannabis phytoligands within SARS-CoV-2 M^pro^

Most of the investigated phytoligands displayed promising binding affinities to SARS-CoV-2 M^pro^ as compared with the reference **(30)** (Table 2). Canniprene **(24)** showed the best binding affinity (-7.02 kcal/mol) followed by cannabigerolic acid monomethyl ether **(3)**, cannabichromene (CBC) **(9)**, cannabigerolic acid (CBGA) **(2)**, cannabinol methyl ether **(16)**, cannflavin B **(23)**, cannabidinodiol (CBND) **(15)** and cannabinolic acid **(14)** which exhibited less binding affinities (-6.98 to -6.67 kcal/mol) but still better than the reference **(30)** that exhibited a binding affinity of -6.66 kcal/mol.

**Table 2.** Results of docking simulations of the studied cannabis phytoligands within SARS-CoV-2 M^pro^.

No.	Name of phytoligands	ΔG^†^ (kcal/mol)	Interactions at the active site of SARS-CoV-2-main protease protease
1	Cannabidiolic acid (CBDA)	-6.12	No interaction
2	Cannabigerolic acid (CBGA)	-6.81	No interaction
3	Cannabigerolic acid monomethyl ether	-6.98	No interaction
4	Cannabigerovarinic acid	-6.57	**Glu166**
5	Cannabigerovarin	-6.62	Gln189
6	Cannabidiol (CBD)	-5.84	No interaction
7	Cannabidivarinic acid	-6.38	Cys145
8	Cannabigerol (CBG)	-4.68	**Asn142**
9	Cannanbichromene (CBC)	-6.87	No interaction
10	Cannabivarin	-6.21	No interaction
11	Δ9-Tetrahydrocannabivarin (Δ^9^-THCV)	-5.96	No interaction
12	Cannabidivarin (CBDV)	-5.89	**Glu166**, Gln189
13	(+)-Cannabichromenic acid	-6.29	No interaction
14	Cannabinolic acid	-6.67	**Glu166**
15	Cannabidinodiol (CBND)	-6.68	**Glu166**, Cys145
16	Cannabinol methyl ether	-6.72	No interaction
17	Cannabinol	-6.12	**Glu166**
18	Cannabicyclolic acid	-4.58	Gln189
19	Cannabicyclol	-4.31	Met165
20	Cannabielsoin A	-5.88	Met165
21	Cannabitriol	-5.47	**Glu166**
22	Cannflavin A	No considerable fitting	No interaction
23	Cannflavin B	-6.71	Cys145, **His163**
24	Canniprene	-7.02	**Glu166**
25	Cannabifuran	-5.97	Cys145, **Glu166**
26	Dehydrocannabifuran	-6.26	Cys145
27	Cannabicitran	-6.21	Met165
28	Cannabiripsol	-4.89	**Glu166**, Gln189
29	Cannabimovone	-6.11	**Asn142**
30	2–Cyclohexyl-*N*-(3-pyridyl)acetamide	-6.66	**Asn142, His163, Glu166**

The key residues involved in the complex formation are listed in bold.

† The ligand–receptor complex binding free energy at RMSD ≤2 Å.

### *In silico* prediction of physicochemical properties, ADME & drug-likeness of cannabis phytoligands

SwissADME online server was employed to predict the most important physicochemical properties that formulate drug-likeness parameters of the most promising phytoligands [17]. The promising phytoligands exhibited drug-like bioavailability according to Veber's [24] and Lipinski's [25] parameters. Among the selected phytoligands, cannabigerovarinic acid **(4)**, cannflavin B **(23)** and canniprene **(24)** were observed to be in full accordance to Lipinski's and Veber's parameters, whereas cannabigerolic acid (CBGA) **(2)**, cannabigerol (CBG) **(8)** and cannabichromene (CBC) **(9)** showed one violation. Additionally, Pre-ADMET program [26] was employed for the calculation of ADME descriptors.

## Discussion

As revealed from molecular docking of the phytoligands within SARS-CoV-2 S protein–ACE2 complex, the most promising phytoligands were cannabigerolic acid (CBGA) **(2)** and its monomethyl ether derivative **(3)**, and cannabigerol **(8)**, belonging to cannabigerol-type phytocannabinoids which were featured by the presence of a linear isoprenyl residue. These phytochemicals were found to be most enriched in cannabis varieties resulted from hybridization [27]. They are nonpsychoactive with low cannabinoid (CB) receptors potency however, they possess powerful antioxidant and anti-inflammatory properties which make them good candidates for managing the inflammatory conditions [28].

Cannabichromene-type phytocannabinoids, represented by cannabichromene **(9)**, possess isoprenyl moiety that is oxidatively fused to the resorcinyl ring. This type was reported to exhibit potent TRPA1 activation, anti-inflammatory and anti-nociceptive actions via the inhibition of cyclooxygenase enzyme and its associated prostaglandins but with no affinity to cannabinoid receptors, i.e., devoid of psychotropic activity [29]. These compounds are more abundant in the vegetative stage of hemp plant than in its reproductive stage [30]. Cannabifuran **(25)**, an oxidatively cyclized analog of cannabinodiol **(15)**, is commonly isolated from aged samples of hashish with no reported biological actions till now.

**Figure 1.**
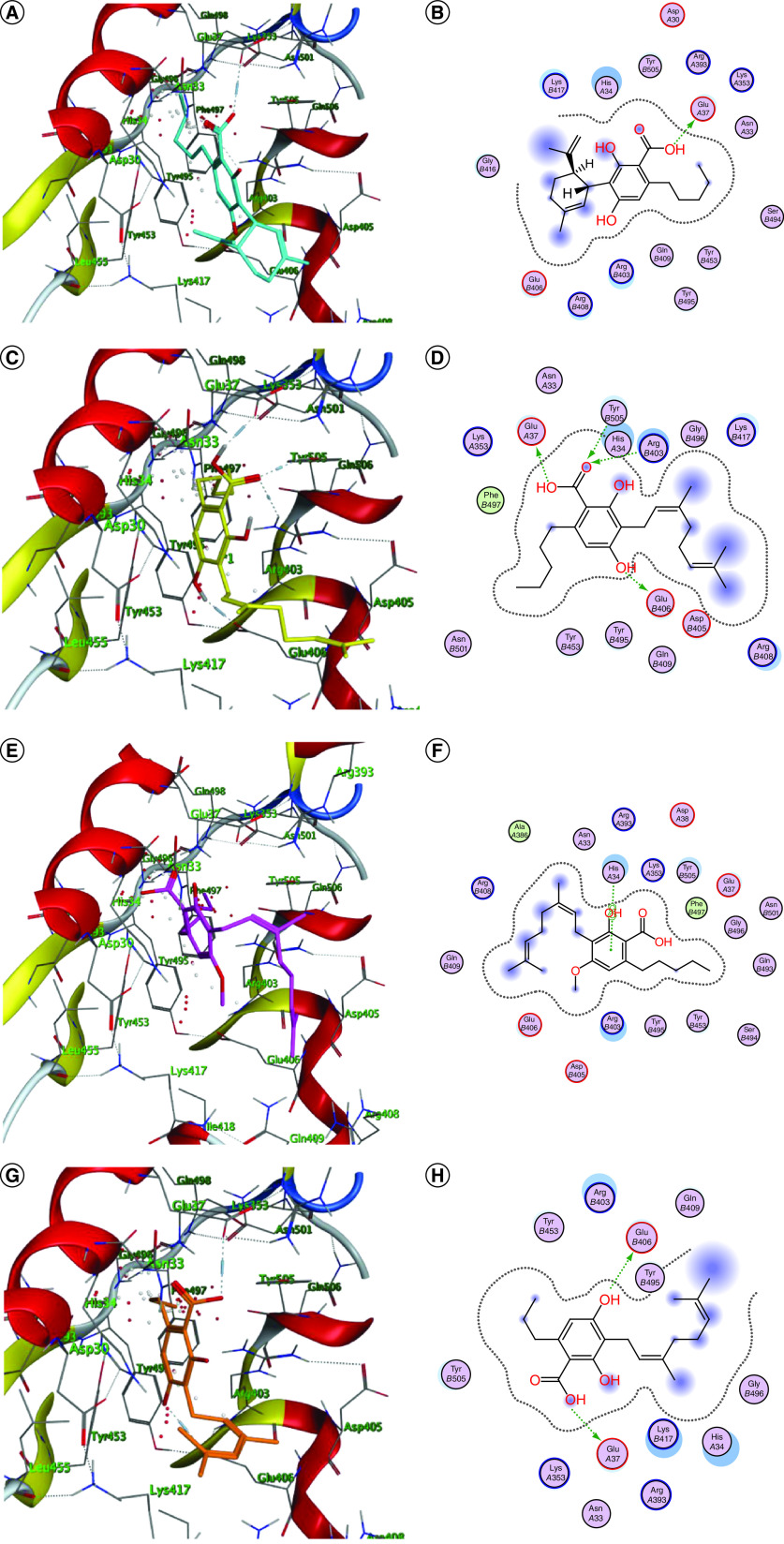

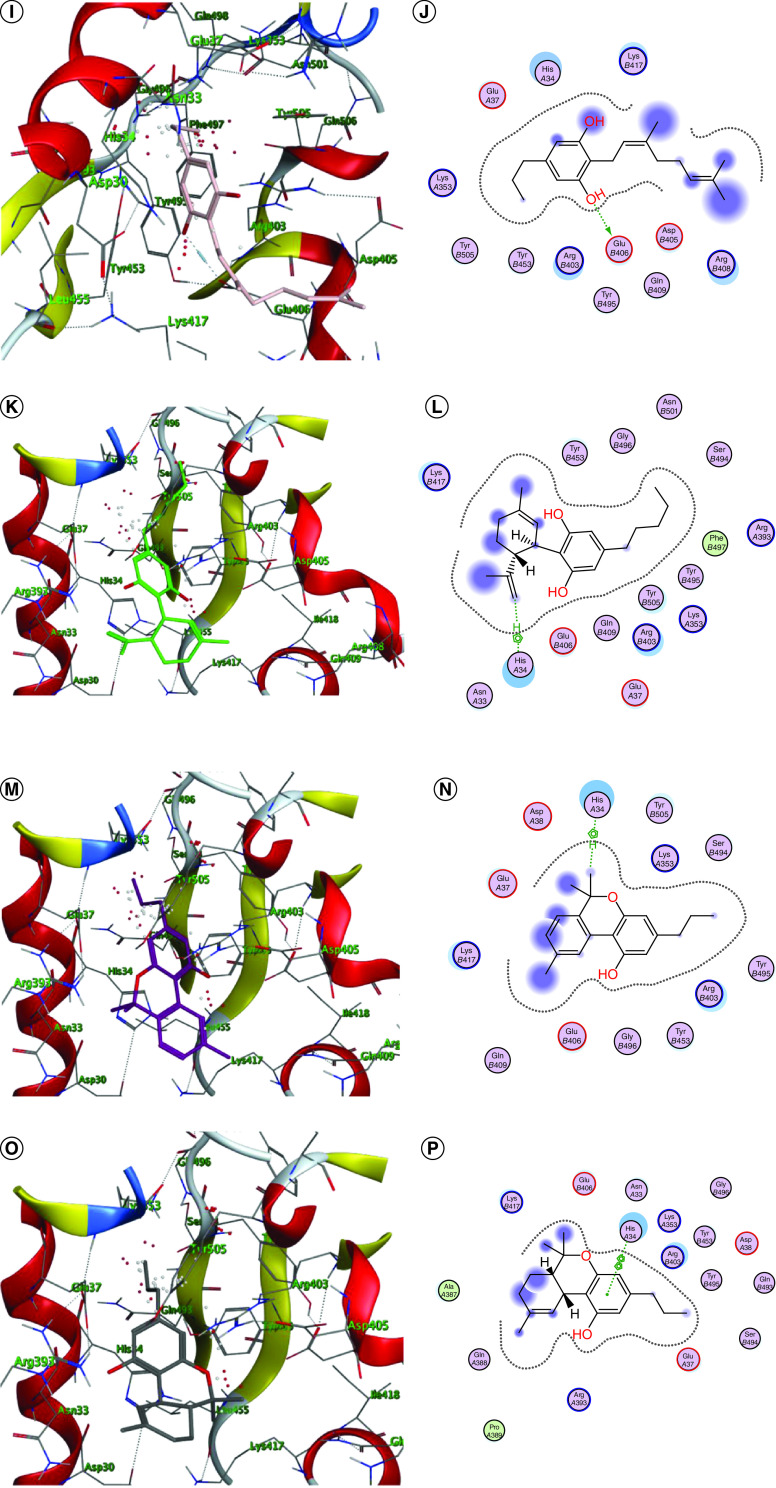

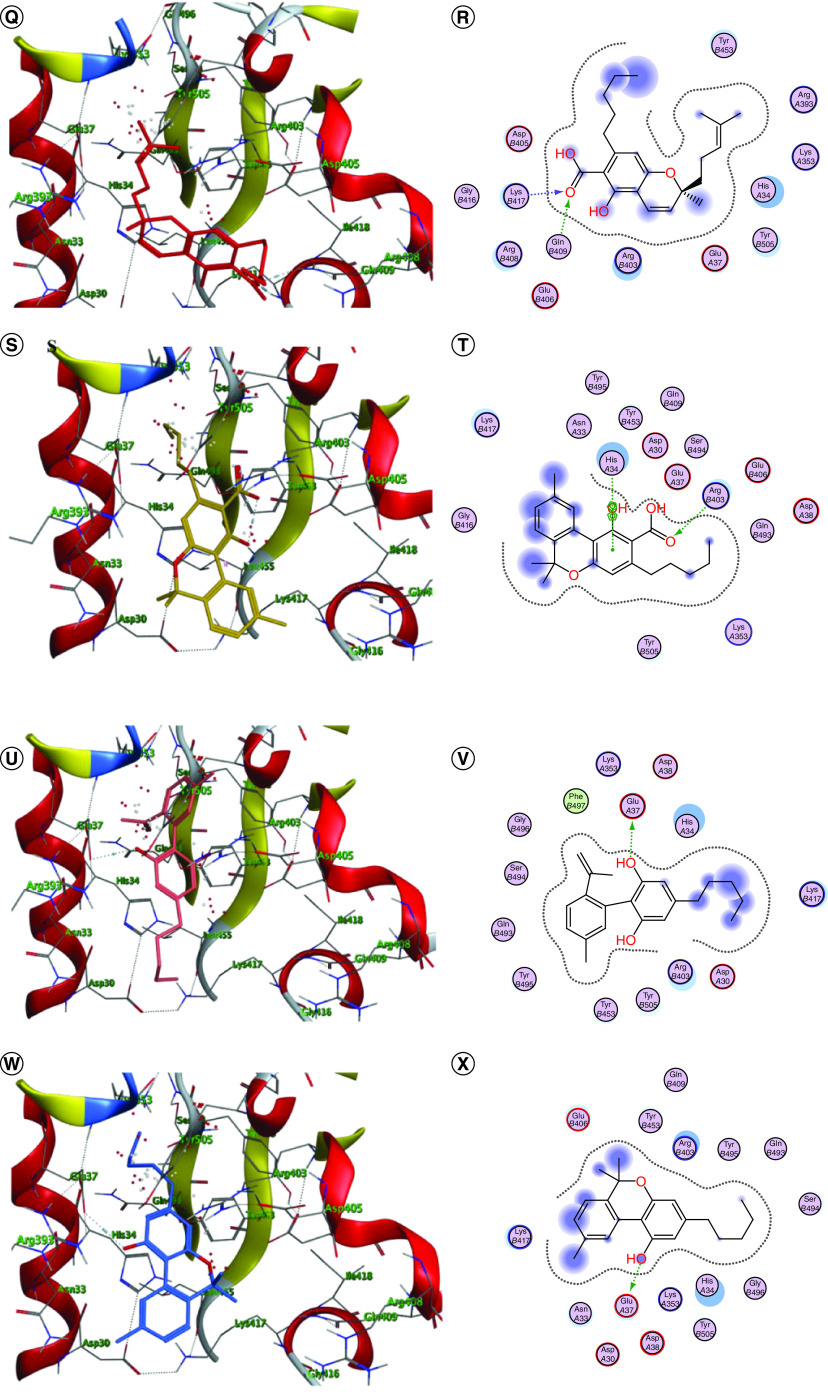

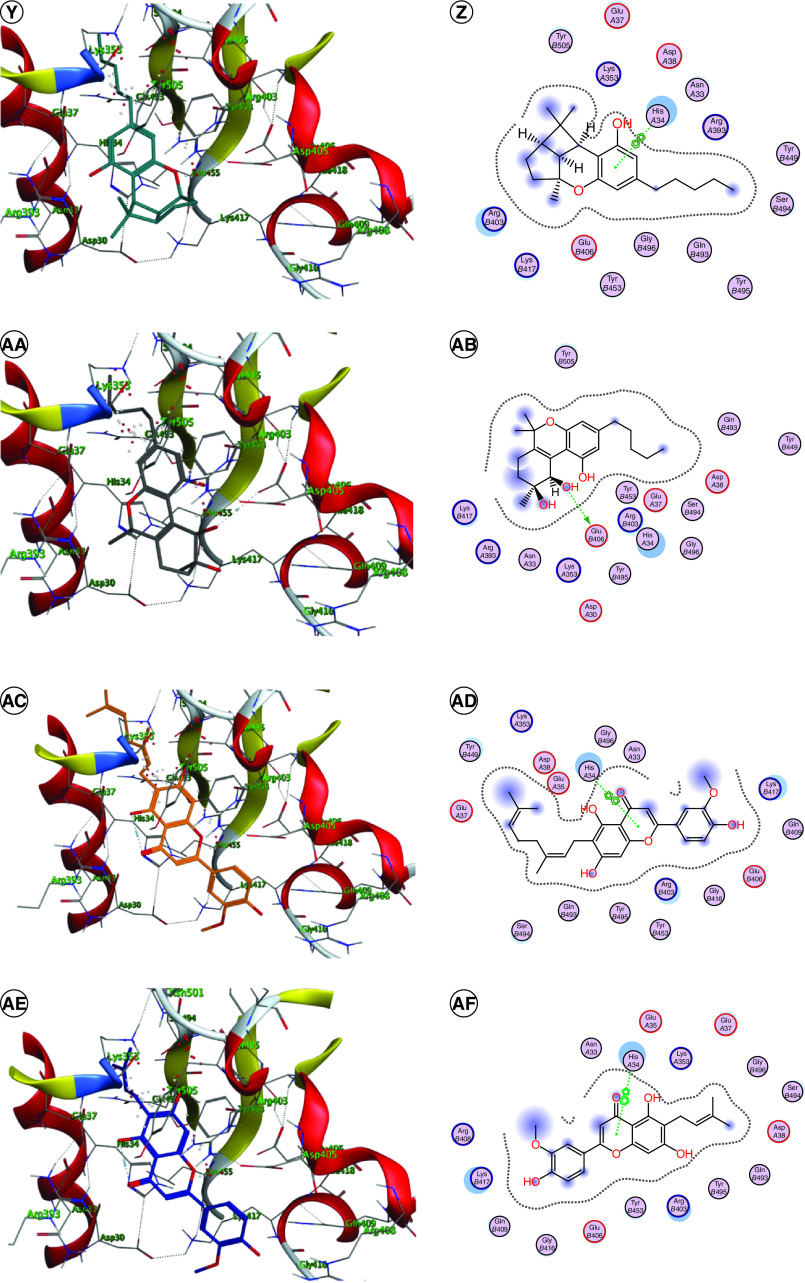

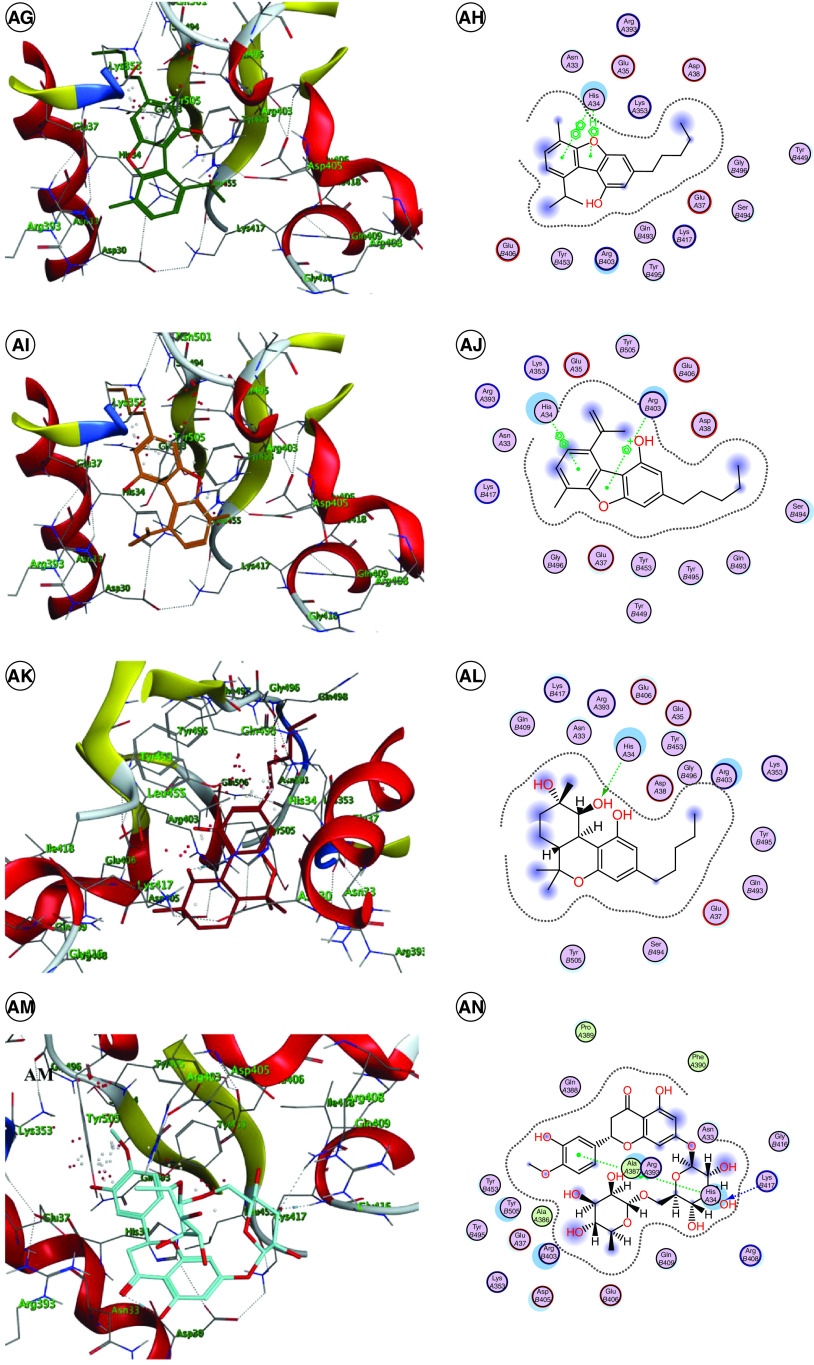
Docking simulation of the studied phytoligands into the binding interface of SARS-CoV-2-CTD in complex with hACE2 (Protein Data Bank ID: 6LZG). **(A & B)** 3D and 2D binding modes of **1** (cyan sticks). **(C & D)** 3D and 2D binding modes of **2** (yellow sticks). **(E & F)** 3D and 2D binding modes of **3** (magenta sticks). **(G & H)** 3D and 2D binding modes of **4** (orange sticks). **(I & J)** 3D and 2D binding modes of **5** (light pink sticks). **(K & L)** 3D and 2D binding modes of **6** (green sticks). **(M & N)** 3D and 2D binding modes of **10** (violet sticks). **(O & P)** 3D and 2D binding modes of **11** (white sticks). **(Q & R)** 3D and 2D binding modes of **13** (red sticks). **(S & T)** 3D and 2D binding modes of **14** (deep yellow sticks). **(U & V)** 3D and 2D binding modes of **15** (pink sticks). **(W & X)** 3D and 2D binding modes of **17** (blue sticks). **(Y & Z)** 3D and 2D binding modes of **19** (deep cyan sticks). **(AA & AB)** 3D and 2D binding modes of **21** (grey sticks). **(AC & AD)** 3D and 2D binding modes of **22** (light brown sticks). **(AE & AF)** 3D and 2D binding modes of **23** (dark blue sticks). **(AG & AH)** 3D and 2D binding modes of **25** (dark green sticks). **(AI & AJ)** 3D and 2D binding modes of **26** (brown sticks). **(AK & AL)** 3D and 2D binding modes of **28** (dark red sticks). **(AM & AN)** 3D and 2D binding modes of **30** (light cyan sticks) in the binding interface of SARS-CoV-2-CTD in complex with hACE2 (PDB ID: 6LZG). The names of the phytoligands **(1–30)** are given in the Materials & methods section.

**Figure 2.**
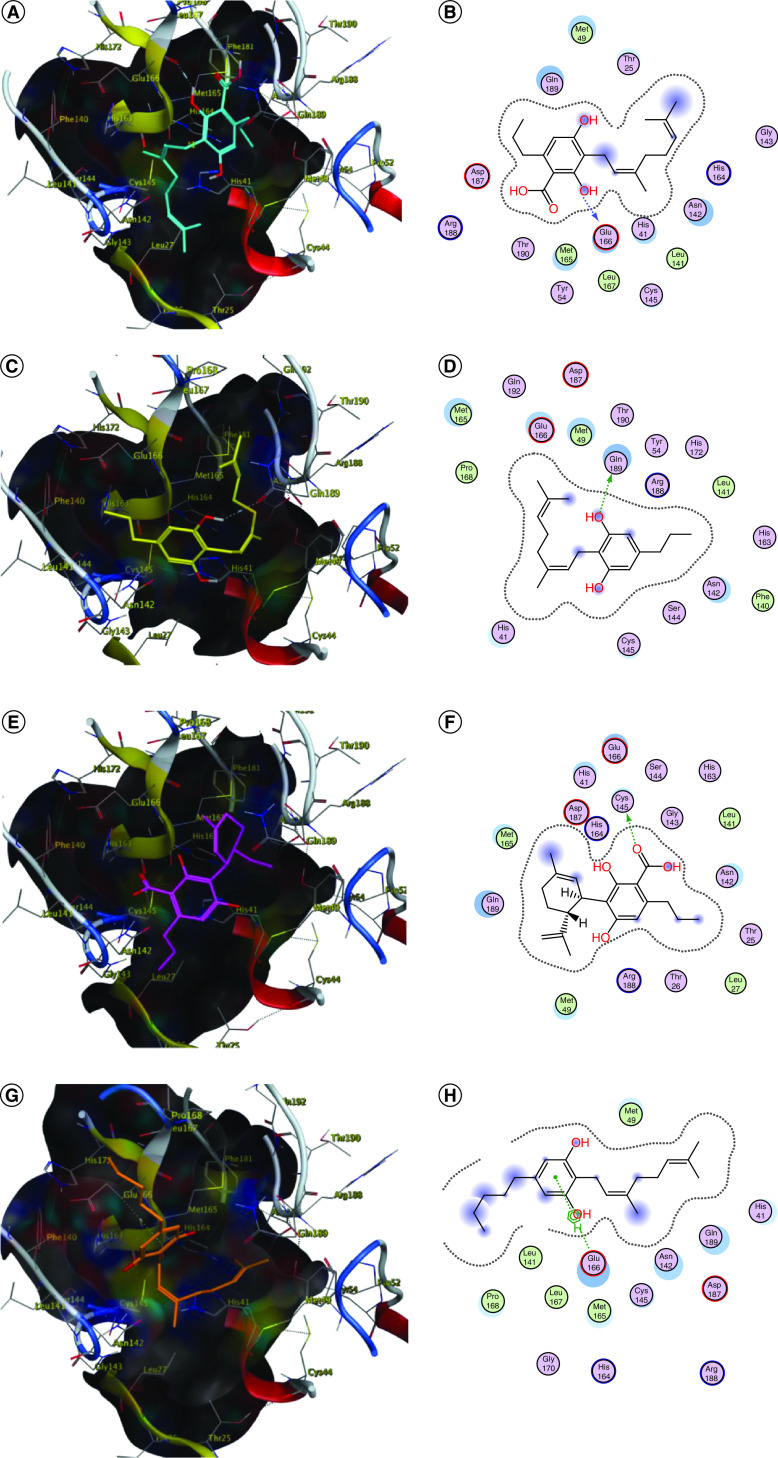

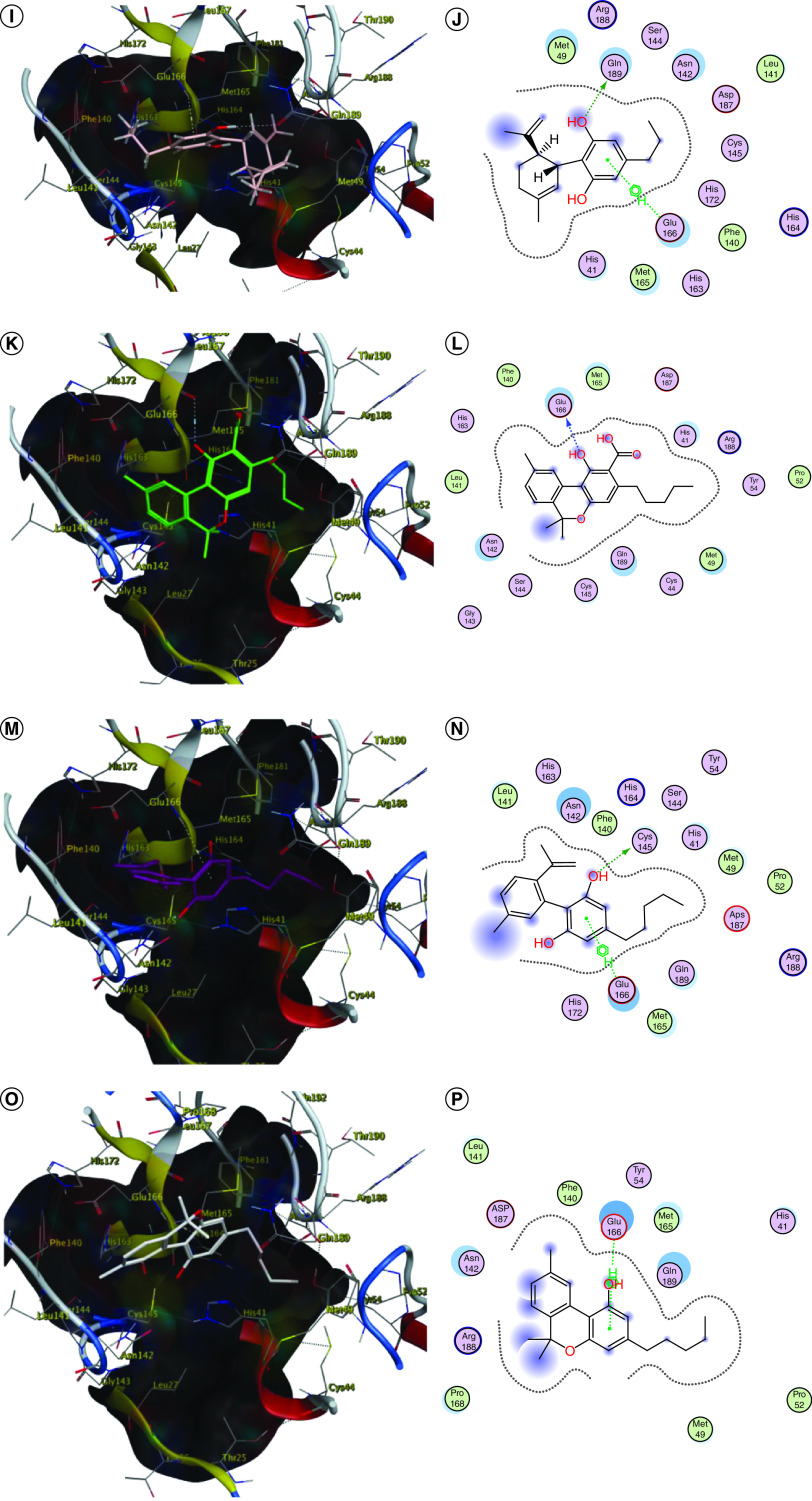

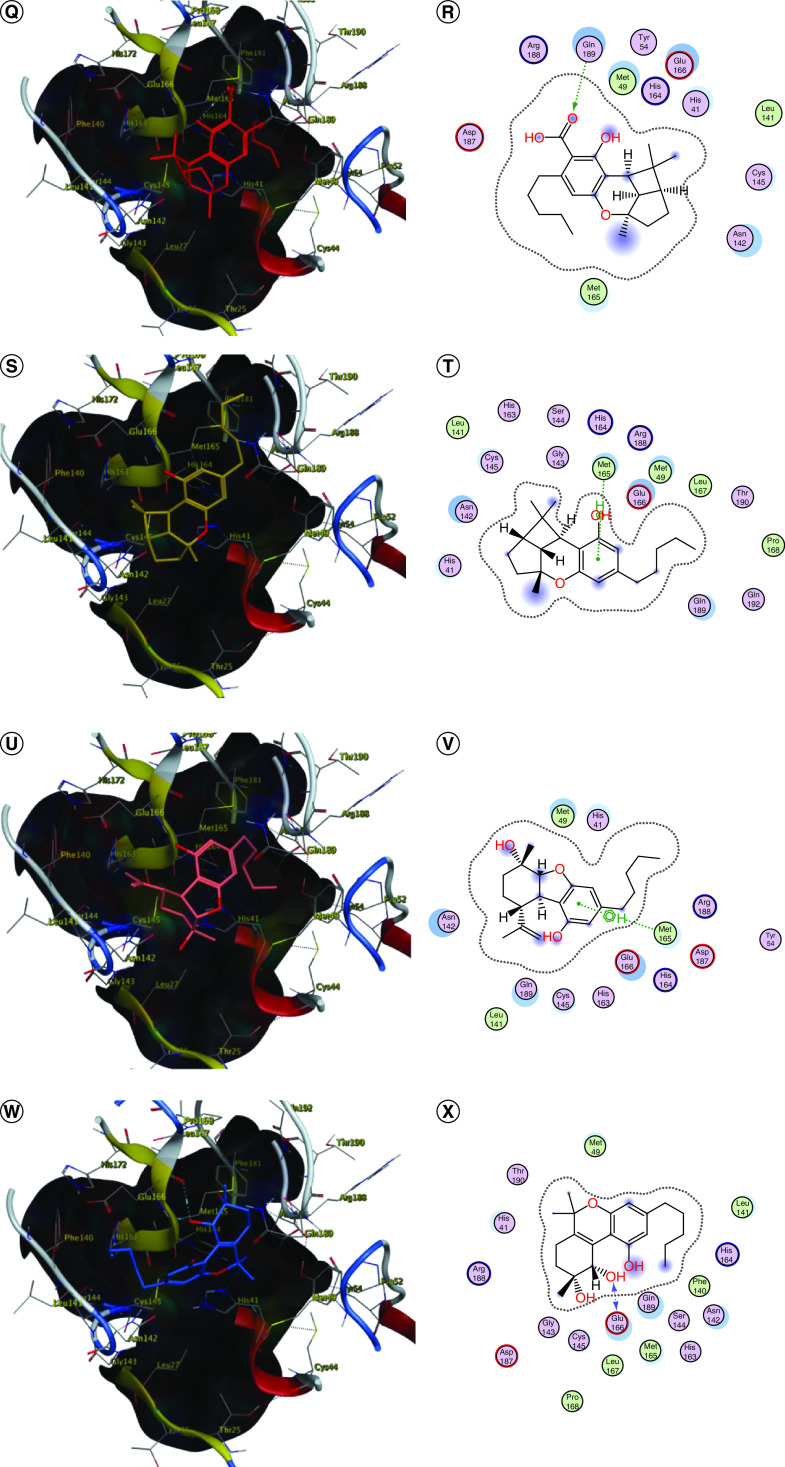

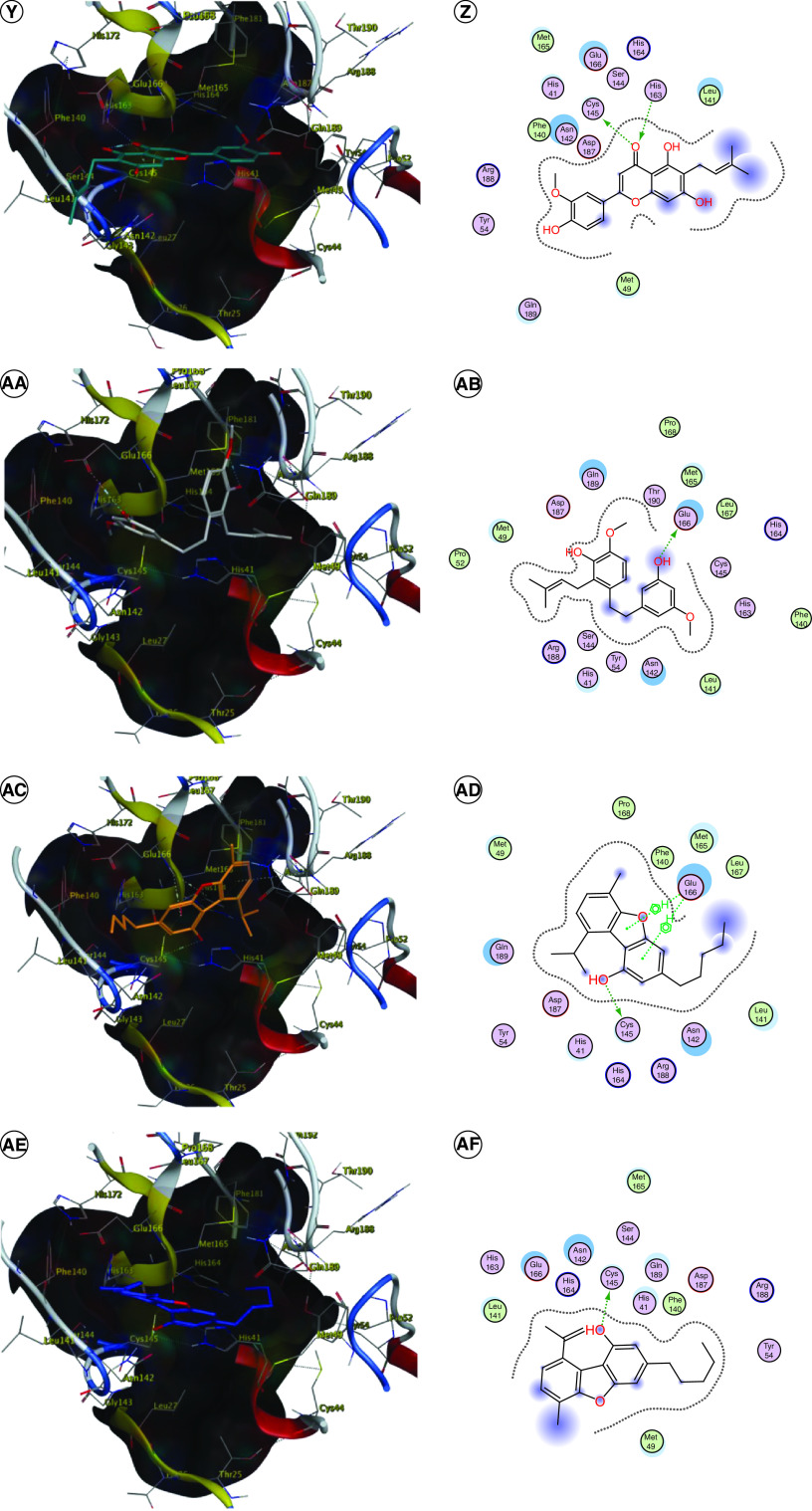

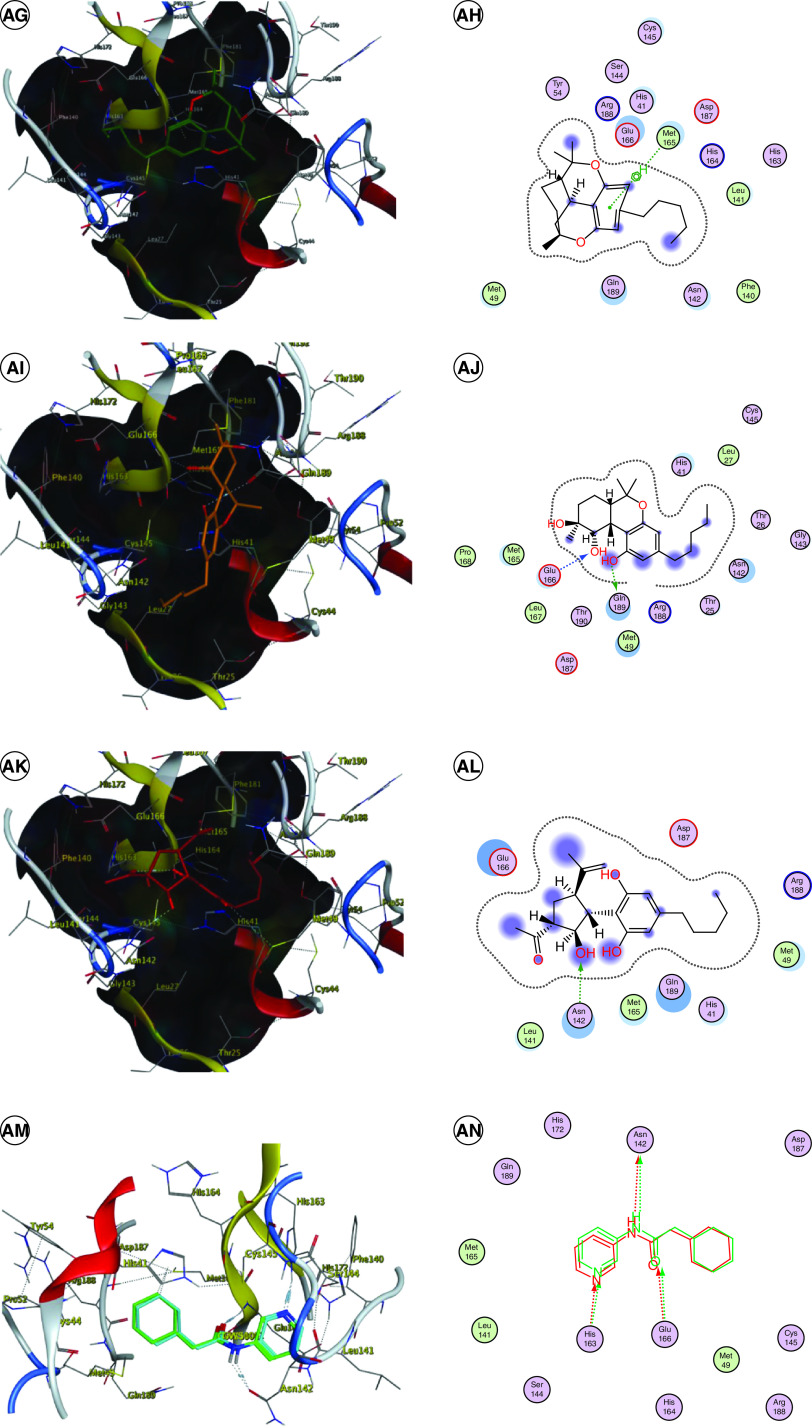
Docking simulation of the studied phytoligands into the active site of SARS-CoV-2-main protease (Protein Data Bank ID: 5R84). **(A & B)** 3D and 2D binding modes of **4** (cyan sticks). **(C & D)** 3D and 2D binding modes of 5 (yellow sticks). **(E & F)** 3D and 2D binding modes of **7** (magenta sticks). **(G & H)** 3D and 2D binding modes of **8** (orange sticks). **(I & J)** 3D and 2D binding modes of **12** (light pink sticks). **(K & L)** 3D and 2D binding modes of **14** (green sticks). **(M & N)** 3D and 2D binding modes of **15** (violet sticks). **(O & P)** 3D and 2D binding modes of **17** (white sticks). **(Q & R)** 3D and 2D binding modes of **18** (red sticks). **(S & T)** 3D and 2D binding modes of **19** (deep yellow sticks). **(U & V)** 3D and 2D binding modes of **20** (pink sticks). **(W & X)** 3D and 2D binding modes of **21** (blue sticks). **(Y & Z)** 3D and 2D binding modes of **23** (deep cyan sticks). **(AA & AB)** 3D and 2D binding modes of **24** (grey sticks). **(AC & AD)** 3D and 2D binding modes of **25** (orange sticks). **(AE & AF)** 3D and 2D binding modes of **26** (dark blue sticks). **(AG & AH)** 3D and 2D binding modes of **27** (dark green sticks). **(AI & AJ)** 3D and 2D binding modes of **28** (brown sticks). **(AK & AL)** 3D and 2D binding modes of **29** (dark red sticks). **(AM & AN)** Overlay of the docked (light cyan sticks) and the co-crystallized inhibitor 2-cyclohexyl-*N*-(3-pyridyl)acetamide **30** (green sticks) in 3D and 2D binding modes, in the active site of SARS-CoV-2-main protease (Protein Data Bank ID: 5R84). The names of the phytoligands **(1–30)** are given in the Materials & methods section.

Most of the promising phytoligands were able to accommodate into the interface and interact with the key aminoacids (Figures 1 & 2). Accordingly, they can destabilize or halt the virus–receptor engagement which is commonly dominated by polar contacts mediated by these key hydrophilic aminoacid residues [13]. Cannabigerolic acid monomethyl ether **(3)**, cannabidiol (CBD) **(6)**, cannabivarin **(10)**, Δ9-tetrahydrocannabivarin (Δ^9^-THCV) **(11)**, cannabinolic acid **(14)**, cannabicyclol **(19)**, cannflavin A & B **(22 & 23)**, cannabifuran **(25)**, dehydrocannabifuran **(26)** and cannabiripsol **(28)** showed interactions with His34 of the hACE-2 involved in the complex formation (Figure 1), whereas (+)-cannabichromenic acid **(13)** interacted with Lys417 which is the key residue of the SARS-CoV-2 CTD but did not exhibit any interactions with the hACE-2 side (Figure 1Q & R).

Cannabidiol **(6)** is a major phytocannabinoid in fiber hemp formed by spontaneous decarboxylation of its acidic form by the act of light and heat upon cannabis ageing. Despite having structural similarity with Δ^9^-THC, it exhibits a distinct pharmacological profile and lacks any psychoactive properties with a very low affinity for cannabinoids receptors. Cannflavins A and B **(22 & 23)**, methylated isoprenoid flavones, are two of unique *C. sativa* flavonoids with a well-reported anti-inflammatory action via the inhibition of 5-Lipoxygenase (5-LO) and prostaglandin E 2 synthase (mPGES-1) [9].

ACE2 inhibition was reported as being unfavorable in COVID-19 patients due to the consequent decrease in the production of angiotensin 1–7, that possess anti-inflammatory, antifibrotic and vasodilatory actions via the Mas receptor [31]. The protective role of ACE2 was revealed in animal models of acute respiratory distress syndrome [32]. Patients who administer angiotensin-II inhibitors (ACE1) was reported to suffer from severe symptoms with a higher mortality rate than their counterparts who did not take these medications [33]. Thus, ligands not interacting with hACE2 side such as (+)-cannabichromenic acid **(13)** will be considered promising anti-SARS drug leads with a preventive potential.

Canniprene **(24)**, a unique dihydrostilbenoid to *C. sativa*, recorded the highest binding affinities to SARS-CoV-2 M^pro^, which possesses anti-inflammatory action via the inhibition of pro-inflammatory eicosanoids production. Canniprene was reported to exhibit more potent action than cannflavin A as 5-LO inhibitors, however, it is less effective in inhibiting mPGES-1 [34].

Moderate fitting was observed in the rest of the cannabis phytoligands (ranging from -6.62 to -4.31 kcal/mol). Most of the phytoligands were able to accommodate into the active site and showed strong interactions with the key aminoacids (Figure 1). Cannabigerovarinic acid **(4)**, cannabinolic acid **(14)**, cannabinol **(17)**, cannabitriol **(21)** and canniprene **(24)** interacted with Glu166 that is involved in the reference inhibitor–active site complex. Additional interactions were observed in the phytolignds, i.e., cannabidivarin (CBDV) **(12)** and cannabiripsol **(28)** with Glu189, however, phytoligands, i.e., cannabidinodiol (CBND) **(15)** and cannabifuran **(25)** exhibited interactions with Cys145. Cannabicyclol **(19)**, cannabielsoin A **(20)** and cannabicitran **(27)** showed interactions with the neighboring Met165. Cannflavin B **(23)** interacted with the active site through its key residue His163, whereas cannabigerol (CBG) **(8)** and cannabimovone **(29)** interacted through Asn142. A comparative display of the energy binding value of ΔG (-kcal/mol) of Spike–ACE2 complex and M^pro^ with the studied cannabis phytoligands is illustrated in Figure 2.

All the investigated phytoligands displayed acceptable aqueous solubility with canniprene **(24)** appearing at the top of the list with an excellent intestinal absorption (>90%), medium to high BBB penetration, moderate permeability through CaCo-2 cells model and low permeability through MDCK ones (Table 3). Most of the phytoligands were predicted to possess moderate to high plasma proteins binding profiles and expected to be more bound to plasma proteins (PPB ≈ 96–100%). The investigated phytoligands were also predicted to be incapable of inhibiting cytochromes P450 2D6 (CYP2D6) but not CYP3A4, except cannabigerolic acid monomethyl ether **(3)** that was devoid of inhibiting both CYP2D6 and CYP3A4 (Table 3).

Indeed, oropharynx and nasopharynx are the main entry ports for SARS-CoV-2 as well as sources of transmission. Both saliva and nasopharyngeal secretions harbor a significant viral load in asymptomatic or presymptomatic virus carriers, and hence playing crucial role in both the pathogenicity and the transmission of SARS-CoV-2. Accordingly, an antiviral oral and nasopharyngeal rinses can be considered as one of the most efficient intervention in combating SARS-CoV-2 transmission [35]. Previous studies reported for the efficacy of mouthwashes in inhibiting viruses such as HIV, herpes simplex virus (HSV) and Middle East respiratory syndrome coronavirus (MERSCoV) [36–38]. Since, canniprene **(24)** possessed the best optimal ADMET profile, among the studied phytoligands, in addition to its most M^pro^ inhibitory action, this phytoligand might serve as promising drug candidate to be administered orally as being devoid of any psychoactivity.

Cannabigerol-type phytocannabinoids, i.e., cannabigerolic acid **(2)**, cannabigerol **(8)** and its acid methyl ether **(3)**, possessed the most potent inhibitory action on the viral entry machinery in our *in silico* modeling study which makes them promising candidates to be administered via topical application in the form of mouthwash/gargles or intranasally. Cannabigerolic acid monomethyl ether **(3)** was revealed to exhibit a dual promising inhibitory action against both the viral entry and the replication machinery. However, the efficacy and tolerability of the identified phytoligands need to be confirmed for its effective integration into the clinical practice. Table 3.

**Table 3.** Computational physicochemical, ADMET and drug-likeness parameters of the selected phytochemicals.

Compound no.	Physiochemical parameters							ADME							Bioavailability and drug-likeness	
	LogP^†^	M.Wt	HBA	HBD	NROTB	TPSA^††^	S (mg/L)	HIA^§^	PPB^¶^	BBB^#^	Caco2^‡‡^	MDCK^‡^	CYP3A4 inhibitor	CYP2D6 inhibitor	Lipiniski^§§^	Veber^¶¶^
**2**	4.19	360.49	4	3	10	77.76	1.07	93.59	100	6.57	19.45	10.54	Yes	None	1 violation; MLOGP >4.15	Yes
**3**	4.40	374.51	4	2	11	66.76	0.50	96.19	99.88	6.95	23.47	2.62	Non	None	1 violation; MLOGP >4.15	1 violation; Rotors >10
**4**	3.74	332.43	4	3	8	77.76	5.92	93.24	100	4.53	20.23	3.82	Yes	None	Yes	Yes
**8**	4.70	316.48	2	2	9	40.46	0.18	93.53	100	17.70	45.92	49.42	Yes	None	1 violation; MLOGP >4.15	Yes
**9**	4.31	314.46	2	1	7	29.46	0.08	97.63	100	16.28	25.53	27.14	Yes	None	1 violation; MLOGP >4.15	Yes
**23**	1.31	368.38	6	3	4	100.13	2.46	90.89	95.64	0.66	7.80	0.22	Yes	None	Yes	Yes
**24**	3.34	342.43	4	2	7	58.92	21.91	93.83	100	6.80	33.86	0.12	Yes	None	Yes	Yes

BBB: Blood-brain barrier penetration; Hydrogen blood acceptor; HBD: Hyrdogen blood donor; M.Wt: Molecular weight; NROTB: Number of rotatable bonds; PPB: Plasma protein binding; S: Aqueous solubility (mg/L).

† Log P: logarithm of compound partition coefficient between n-octanol and water.

‡ MDCK: permeability through Madin-Darby Canin kidney cells. P_MDCK_ values <25 nm/sec (low permeability), values ≈25–500 nm/sec (medium permeability) and values >500 nm/sec (high permeability) [42].

§ HIA: human intestinal absorption. HIA values <20% (poorly absorbed), values ≈20–70% (moderately absorbed) and values >70% (well absorbed) [39].

¶ PPB values <90% (poorly bound) and values >90% (strongly bound) [18].

# BBB values <0.1 (low CNS absorption), values ≈0.1–2 (medium CNS absorption) and values >2 (high CNS absorption [40].

†† TPSA: polar surface area. Drug-like TPSA <140–150 A^2^.

‡‡ Caco2: permeability through cells derived from human colon adenocarcinoma. P_Caco2_ values <4 nm/sec (low permeability), values ≈4–70 nm/sec (medium permeability) and values >70 nm/sec (high permeability) [41–43].

§§ Lipinski rule: log P ≤ 5, M.Wt ≤500 Da, HBA ≤10 and HBD ≤5 [26].

¶¶ Veber rule: NROTB ≤10 and TPSA ≤140 [25].

## Conclusion

**Figure 3.**
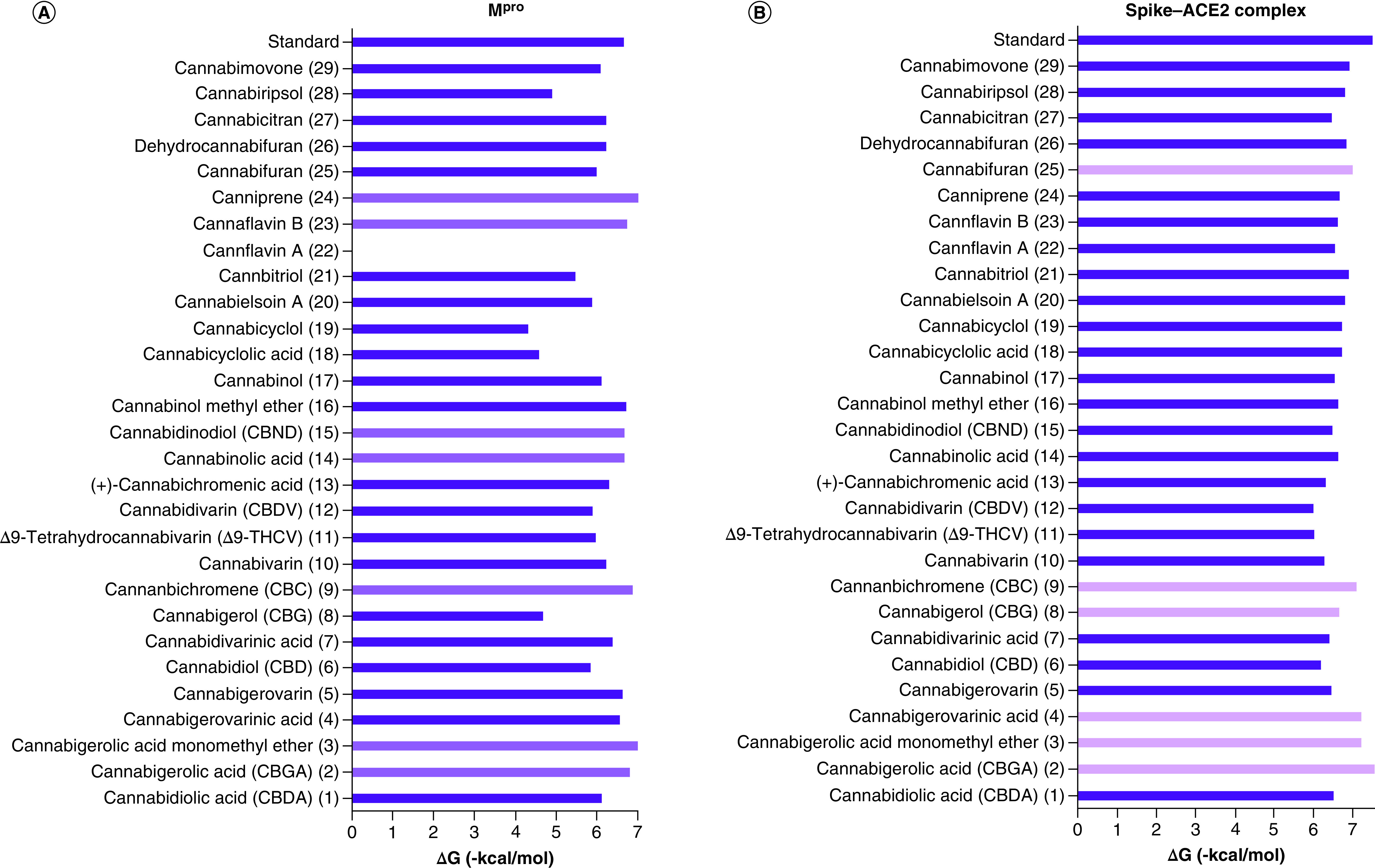
Histograms showing the energy binding value of ΔG (-kcal/mol) of Spike-ACE2 complex and M^pro^ with the studied cannabis phytoligands.

There is an urgency in finding new anti-SARS therapies with an added prophylactic value to be employed side by side to the developed vaccines for an efficient curtailment of COVID-19. The current study posed new drug leads from cannabis with low toxicity and *in silico* dual inhibitory action against viral entry and replication machinery that is essential for a more efficient management of COVID-19. The phytoligands ‘cannabigerolic acid **(2)**, cannabigerolic acid monomethyl ether **(3)** and cannabichromene **(9)**’ possessed the highest inhibitory potential against both viral entry and replication machineries as illustrated in the comparative histograms shown in Figure 3. It seems crucial now to develop dosage forms for local application in the oral cavity to halt the SARS-CoV-2 load for prophylaxis [44]. Intranasal delivery of the proposed drug leads is then suggested as an additional option to minimize COVID-19 propagation and this represents an important area in viral respiratory diseases research. However, this is worthy of further exploration for the sake of identifying optimal parameters for intranasal delivery.

The remarkable *in silico* anti-SARS entry and replication machinery potential of cannabis phytoligands has shed the light on revisiting the cannabis bright side. This further stimulates the exploration of the possible biological actions of other cannabis phytochemicals than the psychoactive cannabinoid motif.

**Summary points**

- *In silico* screening of 29 cannabis phytoligands against SARS spike-ACE2 complex and M^pro^.
- Cannabigerolic acid and cannabigerol were revealed as inhibitors of SARS-CoV-hACE2 complex.
- Canniprene and cannabigerolic methyl ether possessed anti-M^pro^ activity.
- The identified phytoligands could serve as anti-SARS therapies with an added prophylactic value.
